# Development of a theory-based, peer support intervention to promote weight loss among Latina immigrants

**DOI:** 10.1186/s40608-015-0047-3

**Published:** 2015-03-19

**Authors:** Andrea L Cherrington, Amanda L Willig, April A Agne, M Cecilia Fowler, Gareth R Dutton, Isabel C Scarinci

**Affiliations:** Division of Preventive Medicine, School of Medicine, University of Alabama at Birmingham, MT 612, 1720 2nd Avenue South, Birmingham, Alabama 35294 USA; Division of Infectious Diseases, School of Medicine, University of Alabama at Birmingham, BBRD 207, 1720 2nd Avenue South, Birmingham, Alabama 35294 USA

**Keywords:** Obesity, Latino, Peer support, Community-based participatory research, Community Health Workers, Diabetes prevention

## Abstract

**Background:**

Obesity rates are disproportionately high among Latinas living in the United States. Few community-based weight management studies have focused on Latina immigrants living in emerging Latino communities. The purpose of this study was to develop and pilot test a theory-based, promotora-delivered, peer support weight loss intervention for Latina immigrants to be administered in a community setting.

We employed participatory methods to develop an 8-week program grounded in self-determination theory. Overweight Latina immigrants were recruited to participate in a quasi-experimental pilot study. Data collected pre and post-intervention included height, weight, fasting lipids, glucose, dietary practices, physical activity and depressive symptoms.

**Results:**

Twenty-two women completed the intervention. Mean age was 36, mean time in the U.S. was 12 years; the majority was from Mexico. Mean BMI was 33; 68% had a family history of diabetes. The intervention resulted in statistically significant weight loss (mean 2.1 kg, SD 2.6, p < 0.001); mean change in weight remained significant when compared with that of a historical control group (-2.1 kg vs 1.10 kg, p < 0.01) but was attenuated at 6 months. Levels of moderate physical activity increased significantly (p < 0.05) and dietary practices improved (p < 0.01) and remained significant at 6 months. Notably, depressive symptoms also improved (p = <0.001).

**Conclusions:**

This theory-based, promotora-delivered intervention resulted in significant weight loss among a sample of Latina immigrants at 8 weeks. Future studies are needed to test the impact of an extended peer support intervention on long-term weight management.

**Trial registration:**

National Clinical Trials: NCT02344212. Registered 21 January 2015.

## Background

Obesity is a significant risk factor for diabetes, and modest weight loss is a key factor for diabetes prevention [1]. Obesity rates among Latinos are disproportionately high and are especially pronounced among women [2]. For example, 42% of Mexican American women are obese compared to 30% of non-Hispanic white women [3]. While Latino immigrants often arrive in the United States (U.S.) at a healthy weight, time living in the U.S., and changes in lifestyle, diet, and physical activity, are all associated with weight gain [4-6]. Without a significant shift in current trends, it is predicted that the adult Latino population will have an over whelming diabetes prevalence at more than 20% by 2031 [7].

Geographically, most Latinos in the U.S. still live in 9 states that have large, long-standing Latino communities, however, the proportion of individuals living in other states has been growing [8]. As of 2010, 25% of Latinos live in states other than those 9, in what may be considered “emerging Latino communities”. Immigrants in newly emerging communities face unique challenges when it comes to health promotion, including underdeveloped social networks and limited access to education and health related resources [9]. To be effective in emerging communities, interventions should give consideration to these unique, contextual challenges; however a recent literature review identified a limited number of weight loss studies in Latino communities and not one was conducted in an emerging community [10]. Given the disproportionate growth rate of Latino immigrants in emerging communities, there is a need for weight loss interventions that are simultaneously evidence-based, as well as culturally- and contextually-sensitive to the needs of these high-risk communities, particularly the need for increased social support. A promotora-delivered peer support intervention may be the ideal way to achieve this goal. Promotoras are trusted, lay individuals from Hispanic/Latino communities who receive targeted training to provide health education and support within their communities [11]. They serve as a bridge between their own community and the health system as well as other social service organizations [12].

Although Latinos now comprise the largest minority group in the U.S., strikingly few weight management studies have focused solely on Latinos and fewer still on Latino immigrants [8]. The multi-site Diabetes Prevention Program (DPP), an intensive lifestyle intervention involving weight loss and physical activity, included 15% Latinos in its sample of 3,234 participants [13]. While this intensive intervention reduced the risk of developing diabetes by 58% in high-risk individuals, Latinos in this study were English speaking and presumably more highly acculturated than most recent immigrants. A systematic review published in 2013 [10] identified 7 randomized controlled trials examining the effectiveness of weight-loss interventions among adult Latinos living in the U.S. [14-20]. Most interventions were delivered by dieticians, nurses or other health professional; only 2 were promotora-led, a third was led by a promotora/nurse team. Results from these 2 peer-based studies are promising; however they are limited by small samples sizes (range 18 to 72). None of the trials were conducted in what might be considered emerging communities.

We developed a theory-based, promotora-delivered intervention to promote weight loss among immigrants in an emerging Latino community in Alabama. The intervention, entitled *ESENCIAL Para Vivir* (Essential for Life), incorporates the cultural beliefs, attitudes and perspectives of recent, mostly Mexican, Latina immigrants. Grounded in Self-Determination Theory (SDT), the intervention is delivered by a promotora and is designed to promote autonomous motivation for weight-related behaviors by enhancing individuals’ sense of autonomy, competence, and relatedness to others [21]. Below, we briefly describe the development process for this program, intervention content and theoretical underpinnings, and pilot study results.

## Methods

### Research setting

This study took place in Birmingham, Alabama (AL). AL has some of the highest rates of obesity in the nation with over 30% of adults classified as obese (BMI > 30 kg/m^2^). The state has the second highest rate (11.1%) of adults diagnosed with diabetes [22]. Also, AL has one of the fastest growing Latino populations in the nation, with growth rates close to 200% over the past two decades; according to the 2010 census, Latinos comprise 3.6% and 4.1% of the population in Birmingham and Alabama respectively (up from 1.5% in 2000) [8,23]. Even during the recent economic downturn, AL remained second in the nation for growth of the Latino immigrant population.

### Study protocol and procedures

The study protocol and procedures were approved by the University of Alabama at Birmingham’s Institutional Review Board. All participants provided written informed consent in their target language (English or Spanish) prior to commencing study-related measures and procedures. All study procedures comply with the Declaration of Helskini.

### Intervention development

#### Advisory board

An advisory board inclusive of all stakeholders was created to help guide the intervention development process. This board included community members, promotoras, an endocrinologist (from Mexico), a bicultural nutritionist and representative from the health department’s Office of Minority Health, and a behavioral scientist with expertise in community-based methods and Latino health. The board met quarterly to review qualitative results and propose intervention strategies, content, and materials.

#### Focus groups

Formative work included 9 focus groups with community members and 18 semi-structured interviews with managers of peer-led programs and promotora themselves [24-26]. Focus groups revealed that culturally tailored nutrition/lifestyle programs are scarce but welcomed, provided they incorporate traditional foods and customs. Women wanted practical strategies and social support for physical activity and family involvement, including information for children and buy-in from their spouse. Promotoras reported challenges feeling confident in their role and a desire for additional resources to help address medical and health-related topics. Program managers identified issues around ensuring intervention fidelity. Themes identified were used to develop intervention strategies.

#### Peer leader recruitment and training

We recruited one bilingual, bicultural promotora locally through word of mouth. Requirements for hire included good personal communication skills, a driver’s license, and a desire to work in the community. For this intervention, we provided training in topics related to each session as well as basic training in communication and the principles of Motivational Interviewing (MI). MI emphasizes an individual’s control and explores their ambivalence about change, and unlike traditional health education it does not rely on the delivery of untailored advice [27]. MI has been successfully implemented in weight loss interventions and is congruent with SDT and autonomous motivation (described below) [28]. Throughout training, the promotora participated in role-playing and conducted practice sessions to gain confidence with the material.

### Intervention content and delivery

Data collected during the formative phase was used to develop a peer-led eight-week intervention consisting of six group sessions and two individual sessions. In response to the need for spousal and family buy-in, an orientation session and a graduation ceremony were developed and families were invited to attend. Promotoras facilitated small and large group discussions that centered on identifying personal as well as family-level values related to health and well-being [24]. Subsequent sessions were designed to be interactive and included a combination of didactic information about diabetes prevention, healthy nutrition, and physical activity promotion, as well as group and individual activities and discussions (Table 1). In addition to information provided directly by the peer leader, an educational DVD was developed as a teaching tool to deliver brief didactic health education in a fun and informative way. Additionally the DVD functioned to relieve the burden for the promotora to become the “health expert”. For example, myths and misinformation were addressed during a scripted ‘talk show’ with two nutrition experts fielding questions from callers. Questions from participants in previous studies regarding healthy lifestyles were collected and used to develop content for the talk show. A separate physical activity DVD provided a convenient way for women to exercise at home if they felt unsafe in their neighborhood or if they were unable to find child-care.

**Table 1:** **ESENCIAL Para Vivir pilot intervention content and delivery methods**

**Session**	**Focus**	**Content**	**Delivery methods**
1	Diabetes Risk & Prevention	▪ Diabetes Risk and Prevention	DVD
		▪ Personal Values & Your Health	Activity
		▪ Group Exercise	DVD
		▪ My Action Plan – Small Goals Towards Health	Homework
2	Barriers to Healthy Living (Individual)	▪ Barriers to Physical Activity & Healthy Eating	Assessment
		▪ Setting Goals	Activity
3	Bases for Healthy Eating I	▪ The Food Pyramid: A guide to a healthier life	DVD
		▪ The Food Pyramid	Group Activity
		▪ A Rainbow on Our Plate	DVD
		▪ A Rainbow on Your Plate	Activity
		▪ Group Exercise	DVD
		▪ Get more colors on your plate	Homework
4	Bases for Healthy Eating II	▪ My Plate: Choosing how much to eat	Activity
		▪ Food Labels: A guide to eating healthier	DVD
		▪ Food Labels: Identify Key Elements	Activity
		▪ Jose & Julia: What is healthy?	DVD
		▪ Group Exercise	DVD
		▪ Practice reading food labels	Homework
5	Shopping for your Health (Grocery Store)	▪ Making Healthier Choices: Food label comparisons	Activity
		▪ Choosing Healthier Snacks: Is my snack healthy?	Activity
		Treasure Hunt	Activity
6	Ways to Cook Healthier for Life	▪ Cooking with Julia	DVD
		▪ How to make a healthier snack	Activity
		▪ Buying Leaner Meats	Card/Discussion
		▪ Healthier ways to season foods	Card/Discussion
7	Stress Management (Individual)		Activity
		▪ What stresses you?	Discussion
		▪ Ways to avoid/minimize stressful situations	
		▪ Relaxation Techniques	Activity
		How to recognize depression	Card/Discussion
8	Healthy Living for Life	▪ Bases for Healthy Eating (Food Pyramid & Reading Labels)	Review
		▪ Cooking Healthy for Life: Techniques	Review
		▪ Incorporating Physical Activity into Daily Life	Review
		▪ Diabetes Risk & Prevention	Review
		▪ Julia & Jose: Healthy habits for life	DVD

### Theoretical framework of intervention

Self-Determination Theory (SDT) was used as the theoretical foundation for the intervention [21]. SDT distinguishes between motivation that is controlled (i.e., occurs when people act because they feel pressured or compelled to do so) versus autonomous (i.e., occurs when people perceive that reasons for behavior are chosen, emanating from oneself). According to SDT, autonomously motivated behaviors are more likely to be maintained over time [21], while behaviors elicited through controlled motivation are less likely to be maintained when the incentive or threat is removed [21]. There is a growing body of evidence to support this behavior theory, particularly as it relates to lifestyle modification and weight-related behaviors [29,30].

Deci and Ryan, the founders of SDT, identified three basic psychological needs that underlie an individual’s propensity towards autonomous motivation, specifically the need for autonomy, competence and relatedness to others [21]. The *ESENCIAL Para Vivir* intervention was designed to deliver weight loss content through peer support in a way that is autonomy supportive with an overall goal of enhancing autonomous motivation for weight-related behaviors and ultimately promoting weight loss over the long term. Table 2 describes intervention content and peer support strategies as they relate to the psychological needs for autonomy, competence, and relatedness to others.

**Table 2:** **Intervention content and activities related to three psychological needs** ^**†**^

**Psychological needs underlying autonomous motivation**	**Intervention content**	**Peer support strategies**
**Autonomy**	▪ Personalized feedback on current dietary practices and physical activity patterns	✓ Promotora reinforces education & knowledge, including importance of diet, physical activity, and self-monitoring
*Feeling volitional, feeling choice and responsibility for one's behavior*		
	▪ Identification of personal and family values, motivators	Promotora assists with personal goal setting using principles of Motivational Interviewing
	▪ Individualized goal setting	
**Perceived Competence**	Self-monitoring	✓ Promotora reviews goal setting and help participants practice setting SMART^‡^ goals
*Feeling that one can accomplish selected behaviors and reach goals*		
	▪ Activities to practice problem solving	✓ Dietary and physical activity diaries
	▪ Hands-on-learning and activities to practice newly learned skills, such as menu planning and reading labels	Promotora facilitates group discussion of barriers and problem solving skills
	▪ Homework activities to reinforce skills learned in class	✓ Promotora provides ongoing emotional support and encouragement
**Relatedness to Others**	▪ Encourages family discussion of shared values and health related goals	Discuss strategies to identify and reach out to one’s support network
*The need to feel understood, cared for and valued by significant others*		
	Incorporates traditional foods and cultural practices identified through participatory development process	Teach and practice stress management skills
		Buddy system for support and accountability
	▪ Peer Leader provides ongoing emotional support and encouragement	Group support for problem solving and physical activity
		✓ Promotora led monthly support groups

^†^Psychological needs derived from Self-Determination Theory [21].

^‡^SMART = Sustainable, Measureable, Attainable, Realistic, Timely.

### Pilot testing

Following development of the promotora-led intervention, Latina women were recruited to participate in pilot study of the program. Four groups of women (n = 6-10) were recruited from September to December 2009 through a local safety-net hospital, multicultural center, and by word-of-mouth. A bilingual research assistant screened the prospective participants for eligibility and invited them to an enrollment day. Inclusion criteria were foreign born, self-identified as Latina, no history of diagnosed diabetes, fasting blood sugar < 126 mg/dL, and over-weight or obese (BMI > 25 kg/m^2^). Additional exclusion criteria were: any medical condition for which weight loss was contraindicated; a fasting glucose > 126 mg/dL, pregnancy, postpartum less than 6 months, or planning a pregnancy before the end of the study period.

### Data collection procedures and measurement tools

After obtaining written consent, measurement of each participant’s height, weight, fasting glucose, and a rapid lipid panel was completed. Participants also completed a questionnaire assessing demographics, health behaviors, and psychosocial constructs. Data were collected at baseline, following the 8-week intervention, and at a 6-month follow-up. Weight was measured using an electronic scale (Health-O-Meter Professional 349KLX, Health-O-Meter, Boca Raton, FL). Height was measured using a portable stadiometer (Seca 217, Seca, Columbia, MD). A finger stick was completed to obtain fasting glucose (TrueResult Meter, HOMEdiagnostics, Fort Lauderdale, FL) and a rapid lipid panel (CardioChek P.A. Analyzer, CardioChek, Indianopolis, IN). Participants also answered a series of questions on demographics, health status, depressive symptoms, dietary habits, and physical activity. The survey was administered in-person by a trained bilingual/bicultural interviewer in 45 minutes or less.

Depressive symptoms were assessed using the previously validated 8-item *Patient Health Questionnaire* (PHQ-8) [31]. The Spanish version has been previously validated [32]. Dietary practices were assessed by the *Dietary Behavioral Strategies Scale (DBSS)*, a validated tool developed specifically for Mexican immigrants that consists of 30 items measuring dietary behaviors related to diets lower in saturated fat and higher in fiber [33]. The tool was developed to serve as a rapid assessment (checklist) of dietary behaviors and previous studies have demonstrated a high level of correlation between most DBSS items and results obtained via 24-hour recall. Response options range from 1 = never performs this behavior to 4 = almost always performs this behavior. In addition to the DBSS, three 24-hour food recalls were conducted with each participant (2 weekdays, one weekend day). The 24-hour recalls were gathered by bilingual trained interviewers using a standardized multiple-pass interview approach with Nutrition Data System for Research (NDSR), a computer based software application [34]. Physical activity was assessed two ways. Self-reported physical activity was assessed using the *Global Physical Activity Questionnaire* (GPAQ), a validated measure that has been used in a number of different countries, including among Latinos living in the U.S. [35]. Participants were also given an MTI Actigraph accelerometer to wear for 4 days (3 weekday and 1 weekend day; GT1M, ActiGraph Health Services, Pensacola, FL).

For comparison, we obtained data from a medical chart review to create a historical control group. We obtained a list of all Latinas seen at the clinic over the same period as recruitment for the intervention. Using the chronological list, we reviewed every 10^th^ chart, selecting women who were Spanish speaking, overweight or obese without diabetes, not pregnant or post-partum, and who had a second weight recorded between 8-12 weeks from baseline. This resulted in a group of 19 women.

### Statistical analysis

Descriptive statistics of all participants versus participants who completed the intervention were analyzed using *t*-tests for continuous variables and chi-square or Fisher’s exact test for categorical variables. Pre-post intervention data was compared with paired *t*-tests or Fisher’s exact test (depression). All analyses were completed using SAS statistical software version 9.2 (SAS Institute, Cary, NC, 2002) with a significance level established at P < 0.05.

## Results

Of 35 women who initially enrolled in the program, 28 completed the program, and 26 were available for 6-month follow-up for a retention rate of 75%. Twenty-two women had complete biometric data at 8 weeks (4 women had incomplete accelerometer and/or 24-hour food recalls), and 21 women had complete data at 6 months. Analyses are presented for women with complete biometric data. Demographic characteristics of both women who enrolled as well as women who had complete biometric data are summarized in Table 3.

**Table 3:** **Participants’ demographic characteristics, family history of diabetes, weight and body mass index, and depressive symptoms**

	**All Participants (n = 35)**	**Participants with complete data** ^**†**^ **(n=22)**
Demographic characteristics	N(%)	N (%)
Mean Age (SD)	37.7 (8.3)	36.5 (7.5)
Education completed		
Less than High School	27 (77)	16 (73)
Marital status (%)		
Married/Living together as married	28 (80)	19 (86)
Employment (%)		
Full or part time	18 (51)	12 (55)
Health Insurance (%)	5 (14)	3 (14)
Country of origin		
Mexico	30 (86)	18 (82)
El Salvador	3 (9)	2 (9)
Costa Rica	2 (5)	2 (9)
Mean Years in U.S. (SD)	12.6 (5.8)	12.2 (4.9)
Mean Years in A.L. (SD)	9.9 (4.8)	10.5 (4.7)
Self-rated health (%)		
Excellent/good	17 (49)	11 (50)
Fair/poor	18 (51)	11 (50)
Family history of diabetes (%)	24(69)	15 (68)
Child birth weight > 9lbs	13 (38)	8 (38)
Anthropometric measures		
Mean Height (cm; SD)	158.6 (5.8)	159.2 (5.5)
Mean Weight (kg; SD)	82.9 (13.9)	83.3 (14.5)
Mean BMI (SD)	32.9 (4.8)	32.7 (4.7)
Told by MD to lose weight	18 (51)	14 (64)^‡^
Depressive symptoms (%)		
No symptoms	13 (37)	5 (23)
Minimal	16 (46)	13 (59)
Moderate/severe	6 (17)	4 (18)

^†^Participants (n=22) with complete data for baseline, 8 weeks, and 6-month follow-up.

^‡^P < 0.05 for n=35 versus n=22.

Of note, women who reported having been told by their doctor to lose weight were significantly more likely to complete the program.

### Physiologic outcomes

**Figure 1:**
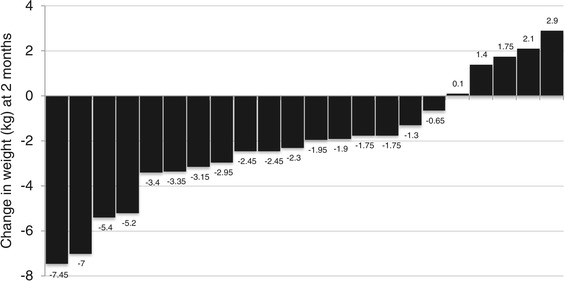
**Individual changes in weight (kg) from baseline to 2-month follow-up (n=22)†.** †Participants (n=26) completed program, only (n=22) had complete data for baseline, 2-month and 6-month follow-up.

After the 8-week intervention, mean weight decreased significantly from 83.3 kg to 81.1 kg (i.e., 4.6-lb weight loss), and mean BMI decreased from 32.7 to 31.8, *p* < 0.001 (Table 4). Eighty percent of participants lost weight after 8 weeks (Figure 1). Mean change in weight was statistically significant when compared with mean change in weight for the historical control group (-2.10 kg vs 1.10 kg, p < 0.01). Intervention weight change at month 6 was not significantly different from baseline, as participants demonstrated modest weight regain following treatment (Table 4). Statistically significant decreases in total cholesterol and LDL were observed at 8 weeks, *p* < 0.001. At month 6, reductions in LDL remained significant, and mean HDL improved significantly at month 6 as well, *p* < 0.03. Paradoxically, mean fasting glucose increased by approximately 6 mg/dL at week 8, but the change was no longer significant at month 6.

**Table 4:** **Change in physiologic and behavioral outcomes at 8 weeks and 6-month follow-up**

	**Baseline** ^**†**^ **(n=22)**	**8-weeks (n=22)**	**p-value**	**6-month** ^**‡**^ **(n=21)**	**p-value**
	**Mean(SD) or N(%)**	**Mean(SD) or N(%)**		**Mean(SD) or N(%)**	
**Physiologic outcomes**					
Weight (kg)	83.3 (14.5)	81.2 (14.0)	0.001	82.2 (14.2)	0.167
BMI	32.7 (4.7)	31.8 (4.8)	<0.001	32.2 (4.9)	0.061
Lipids (mg/dL)					
Total cholesterol	202.9 (55.3)	176.1 (52.5)	<0.001	195.7 (73.8)	0.328
LDL	136.2 (45.3)	97.5 (41.1)	<0.001	103.1 (52.5)	0.002
HDL	45.9 (4.1)	46.6 (14.2)	0.524	52.4 (15.5)	0.023
Fasting glucose (mg/dL)	93.2 (11.2)	99.0 (14.3)	0.007	96.0 (12.4)	0.186
**Behavioral outcomes**					
Dietary practices	2.45 (0.46)	3.14 (0.44)	<0.001	3.09 (0.49)	<0.001
Caloric intake					
Total kcal	2066.4 (731.9)	1580.6 (372.8)	0.006	1460.8 (366.9)	<0.001
%Fat	31 (6)	28 (10)	0.243	28 (7)	0.155
%Carbohydrate	53 (8)	53 (9)	0.984	53 (8)	0.614
%Protein	16 (3)	19 (3)	<0.001	19 (4)	0.002
Moderate/Vigorous PA^¶^, minutes (median)					
Self-report	33.2 (11.8)	80.3 (60.0)	0.004	87.5 (29.3)	0.026
Accelerometer	12.1 (7.7.1)	14.5 (13.3)	0.464	39.5 (16.8)	0.291
**Depressive symptoms (%)**			<0.001		<0.001
No symptoms	5 (23%)	15 (72%)		17 (80%)	
Minimal	13 (59%)	3 (14%)		2 (10%)	
Moderate/severe	4 (18%)	3 (14%)		2 (10%)	

^†^Of 26 participants, 22 participants had complete data for baseline, 8-weeks, and 6-month follow-up.

^‡^One participant was excluded due to pregnancy at 6-month follow-up.

^¶^PA = Physical Activity.

### Behavioral outcomes

At baseline, the average caloric intake was 2,066 kilocalories with 31% of kilocalories coming from fat, 53% coming from carbohydrates and 16% coming from protein (Table 4). At the end of treatment, there was a significant decrease of 486 kcal/day and a significant increase in the percentage of calories coming from protein sources, *p* < 0.006. These changes in caloric and protein intake remained significant at month 6. Healthy dietary practices increased from a mean of 2.45 (SD 0.46) at baseline to 3.14 (SD 0.44) at 8 weeks and the increase persisted at 6 months. Participants’ self-reported minutes spent in moderate to vigorous activity increased at 8 weeks and remained significant at 6 months. While accelerometer data did not demonstrate a significant change in minutes of moderate to vigorous activity at 8 weeks, it did demonstrate a significant increase at 6 months (p < 0.02). Women’s depressive symptoms also improved, with the proportion of those without any depressive symptoms increasing from 23% at baseline to 73% (p < 0.001) at 8 weeks and 80% at 6 months (p < 0.001).

## Discussion

Informed by community-based participatory methods involving local stakeholders and formative work with the target population, we successfully developed and pilot tested a theory-based, promotra-delivered weight loss intervention for Latina immigrants in an emerging community in the Southeastern U.S. The intervention resulted in statistically significant weight losses of 2.0 kg and 80% of women lost weight. Levels of moderate physical activity (PA) increased significantly post-intervention and at the six-month follow-up, though there was significant discrepancy between self-reported PA and more objective measurement via accelerometers. This could relate to an increased awareness about the recommendations for PA leading to more pronounced improvements by self-report than accelerometer, or so-called response bias. Dietary practices also improved and remained significantly better at 6 months. Finally, depressive symptoms improved significantly. This pilot study demonstrates that peer support may be an appropriate mechanism for the provision of evidence-based weight loss strategies in emerging Latino communities.

A number of peer support models exist, and reviews of the evidence suggest there are common, key functions of successful peer support [36]. These include assistance with implementation of daily self-management plans tailored to the specifics of individuals’ lives, provision of ongoing social and emotional support, and linkage to resources [36]. Social support in particular has been identified as a key factor in the success of weight loss and weight loss maintenance, especially for women [37,38]. In addition to its impact on weight and weight related behaviors in this sample, our peer support intervention also produced a dramatic reversal of depressive symptoms. This improvement may be in part due to the increased social support and decreased isolation that peer support interventions have to offer. In contrast to established immigrant receiving communities, immigrants in newly emerging communities face unique challenges when it comes to health promotion, including immature social networks and limited access to education and health related resources [23,39]. Our own qualitative studies have demonstrated that women in these communities are often socially isolated with few outlets for engagement [24]. Thus, peer-delivered interventions may be particularly well suited to the needs of immigrant women in newly emerging Latino communities.

To our knowledge, this is the first community-based weight loss program designed for Latino immigrants rooted in self-determination theory (SDT) [21]. SDT is a particularly appealing behavioral theory for weight management programs because of the potential it holds for promoting long-term behavior change and its intuitive overlap with peer-based programs. To date, maintenance of weight loss beyond the initial intervention phase has proven to be a challenge [40]. According to SDT, behaviors that are autonomously motivated are more likely to be maintained in the long term [21]. An increasing number of studies provide evidence for the link between autonomous motivation and satisfaction of three psychological needs, namely autonomy, perceived competence and relatedness to others [29,41]. To the extent that peer support can facilitate satisfaction of those three needs, it may provide an effective means of delivering evidence and theory based weight loss interventions in community-based settings. Future studies are needed to better elucidate the mechanisms through which peer support exerts its effects, including potential influences on autonomous motivation for weight related behaviors in special populations.

To be effective in diverse communities, behavioral interventions need to include theory based, evidence-based strategies, but they must also resonate with the intended audience. With SDT in place as a theoretical framework, we employed community-based participatory methods to develop an intervention that is culturally relevant for Latina immigrants living in Alabama and emerging communities like it. Qualitative formative work allowed us to identify factors related to surface structure sensitivity, such as preferences regarding language, terminology, presentation style, and traditional food selection, as well as deep structure sensitivity, such as the importance of family, spousal buy-in, social isolation perceived discrimination and the need for social support. By definition, community-based health interventions should be culturally and contextually specific. Our pilot study suggests that SDT may provide a theoretical foundation for community-based weight loss programs that can then be made culturally relevant through the use of participatory methods during the development phase.

Despite the innovation and other strengths of this project, several limitations should be noted. By design, this involved a pilot study with a small number of women. Despite the small sample size, significant effects in a number of clinical, behavioral, and psychosocial outcomes were observed, providing justification for continued investigation of these types of programs with larger samples. Unfortunately, weight loss findings in this study were not significant at 6 months. This result is not surprising since studies have consistently demonstrated that without some structured maintenance program, weight regain is the norm [42,43]. Maintenance sessions were outside the scope of the current pilot but will be an essential component of future investigations examining the longer-term outcomes of culturally adapted interventions such as the one described here. Since the sample included mostly Mexican immigrants, results may not generalize to other Latino populations.

## Conclusion

The approaches applied in this study provide encouraging results regarding the potential utility and efficacy of a promotora-led weight loss intervention for Latina immigrants. Despite a relatively brief (i.e., 8-week) intervention, women demonstrated significant improvements in weight, lipids, dietary intake, physical activity, and depressive symptoms. In addition, some of these improvements were maintained at a six-month follow-up. Results from this pilot study can be used to inform future studies in the areas of measurement (i.e self-reported physical versus objective measurement), intervention content (i.e need for inclusion of maintenance sessions to promote sustained weight loss), as well as target outcomes (i.e. assessment of changes in depressive symptoms in addition to behavioral and physiologic outcomes). Additionally, future studies should assess whether an extended peer support intervention based on self-determination theory can lead to long-term weight management.
